# Treatment completion and challenges in rolling out 12-dose weekly rifapentine plus isoniazid to prevent tuberculosis among people living with HIV and pediatric household contacts in Brazil

**DOI:** 10.36416/1806-3756/e20250125

**Published:** 2025-09-22

**Authors:** Isadora Salles, Jamile Garcia de Oliveira, Alexandra Brito de Souza, Silvia Cohn, Renata Spener-Gomes, Solange Dourado de Andrade, Valeria Saraceni, Solange Cesar Cavalcante, Jeff Weiser, Makaita Gombe, Violet Chihota, Gavin Churchyard, Richard E Chaisson, Marcelo Cordeiro-Santos, Betina Durovni

**Affiliations:** 1. Center for Tuberculosis Research, Johns Hopkins University, Baltimore (MD) USA.; 2. Secretaria Municipal de Saúde do Rio de Janeiro, Rio de Janeiro (RJ) Brasil.; 3. Fundação de Medicina Tropical Dr. Heitor Vieira Dourado, Manaus (AM) Brasil.; 4. Programa de Pós-Graduação em Medicina Tropical, Universidade do Estado do Amazonas, Manaus (AM) Brasil.; 5. Departamento de Saúde Coletiva, Faculdade de Medicina, Universidade Federal do Amazonas, Manaus (AM) Brasil.; 6. Fundação Oswaldo Cruz - Fiocruz - Rio de Janeiro (RJ) Brasil.; 7. The Aurum Institute, Johannesburg, South Africa.; 8. School of Public Health, University of the Witwatersrand, Johannesburg, South Africa.; 9. Division of Infectious Diseases, Vanderbilt University Medical Center, Nashville (TN) USA.; 10. Faculdade de Medicina, Universidade Nilton Lins, Manaus (AM) Brasil.; 11. Universidade Federal do Rio de Janeiro, Rio de Janeiro (RJ) Brasil.

**Keywords:** Tuberculosis, Latent tuberculosis, HIV infections, Isoniazid, Rifapentine, Treatment adherence and compliance

## Abstract

**Objective:**

In July of 2021, the Brazilian National Ministry of Health integrated three months of once-weekly isoniazid plus rifapentine (3HP) into the National Guidelines for Tuberculosis Control as a first-line tuberculosis preventive therapy (TPT) option for people living with HIV (PLHIV) and tuberculosis household contacts (HHCs). As part of the Unitaid-sponsored Increasing Market and Public Health Outcomes through Scaling up Affordable Access Models of Short Course Preventive Therapy for TB project to implement short-course TPT, we evaluated 3HP uptake, completion, and tolerability among PLHIV and pediatric HHCs in Brazil.

**Methods:**

We conducted a multicenter single-arm pragmatic project to roll out 3HP for PLHIV and HHCs in the 2- to 14-year age bracket in the cities of Rio de Janeiro and Manaus, Brazil. Participants were identified, treated, and monitored in accordance with Brazilian national tuberculosis guidelines. De-identified patient-level data on treatment initiation, adverse events, and completion were collected and analyzed.

**Results:**

From October of 2021 to March of 2023, 380 PLHIV (77.6% of whom were male; median age, 40 years [IQR, 31-51]) and 74 HHCs (54.1% of whom were male; median age, 8.6 years [IQR, 5.1-11.8]) were enrolled in the study. Treatment completion rates were 83.7% among PLHIV and 82.4% among HHCs. Completion rates were higher in Rio de Janeiro than in Manaus (PLHIV: 86.0% vs. 79.6%; HHCs: 85.2% vs. 80.9%), although completion of 10 doses was similar (PLHIV: 86.4% vs. 86.1%). Adverse event-related discontinuation was low (PLHIV: 2.4%; HHCs: 2.7%). One person living with HIV developed active tuberculosis during treatment. At six months of follow-up, 99.6% of the PLHIV remained free of tuberculosis.

**Conclusions:**

The 3HP regimen was successfully introduced, had high treatment completion rates, and was well tolerated. Widespread use of 3HP for TPT may accelerate tuberculosis elimination in Brazil.

## INTRODUCTION

Tuberculosis remains a global health crisis and reclaimed its position as the leading cause of death by an infectious agent in 2024, surpassing COVID-19.^1^ Despite relative stability in case numbers over the past two years, 2023 saw 10.8 million tuberculosis cases and 1.25 million deaths, with nearly 13% occurring among people living with HIV (PLHIV). Brazil, one of the 30 high tuberculosis burden countries and among the top 10% globally for tuberculosis/HIV coinfection burden,^2^ reported an estimated 80,012 tuberculosis cases in 2023, with a 9.3% tuberculosis/HIV coinfection rate.^3^ The situation is critical in children under 15 years of age, with 3,409 new cases in 2023, reflecting a rising trend since 2020.^3^

Tuberculosis prevention in PLHIV and pediatric household contacts (HHCs) is crucial for reducing transmission and mortality in these vulnerable populations, given their increased susceptibility to disease progression and the complex diagnostic and treatment challenges that they face.^1^ ^,^ ^4^ ^-^ ^8^

Despite the proven effectiveness of tuberculosis preventive therapy (TPT) in reducing progression of tuberculosis infection to active tuberculosis disease,^9^ fewer than 46% of the nearly 39 million PLHIV, 37% of the 1.56 million eligible HHCs under 5 years of age, and only 15% of the estimated 13 million HHCs of all ages globally received TPT in 2022.^10^ Preventing the progression of tuberculosis infection to active tuberculosis disease is crucial for reducing disease burden and mortality, while assuring progress towards the 2030/2035 targets set by the United Nations General Assembly High-Level Meeting on the Fight against Tuberculosis and the End TB Strategy.^1^

The challenge in scaling up TPT coverage is compounded by adherence issues, particularly with longer treatment regimens. Adherence to longer TPT regimens such as preventive therapy with six months of daily isoniazid has proven challenging.^11^ Isoniazid preventive therapy completion rates are frequently low, with studies indicating that up to 50% of individuals who initiate TPT fail to complete it.^11^ ^-^ ^13^ Nonadherence to isoniazid preventive therapy has been observed to increase over time since TPT initiation.^12^ Short-course TPT regimens, such as three months of once-weekly isoniazid plus rifapentine (3HP), are a promising alternative, with shorter duration and low toxicity levels as incentives for treatment completion, highlighting the relationship between shorter duration and improved adherence.^14^ ^-^ ^16^

Since 2018, the WHO has recommended 3HP consisting of 12 once-weekly doses of rifapentine and isoniazid for children over 2 years of age and adults, including those with HIV, as an alternative to longer regimens.^17^ The Brazilian National Ministry of Health sanctioned the inclusion of the 3HP regimen at the end of 2020.^18^ In July of 2021, the Brazilian National Ministry of Health integrated 3HP into the National Guidelines for Tuberculosis Control as a first-line TPT option for PLHIV and tuberculosis contacts, with free distribution in the Brazilian Unified Health Care System starting in the last quarter of 2021. As part of the Unitaid-sponsored Increasing Market and Public Health Outcomes through Scaling up Affordable Access Models of Short Course Preventive Therapy for TB (IMPAACT4TB) project to implement short-course TPT, we evaluated 3HP uptake, treatment completion, and tolerability in PLHIV and pediatric HHCs in Brazil during its nationwide rollout. 

## METHODS

We conducted a multicenter single-arm pragmatic project to roll out 3HP for PLHIV and pediatric HHCs in the cities of Rio de Janeiro and Manaus, Brazil. Although 3HP was incorporated into the National Guidelines for Tuberculosis Control as a frontline TPT choice in July of 2021, regulatory delays slowed the importation of rifapentine, postponing 3HP implementation. Rifapentine (Priftin^®^; Sanofi S.A., Paris, France) arrived in Brazil in September of 2021, and study enrollment began in October of 2021 with the incorporation of 3HP into the Brazilian Unified Health Care System. 

**Figure 1:**
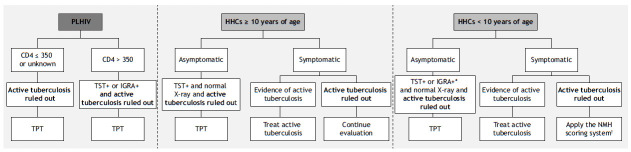
Brazilian national guidelines on tuberculosis preventive therapy (TPT) for people living with HIV (PLHIV) and household contacts (HHCs). TST: tuberculin skin test; and IGRA: interferon-gamma release assay. *Only in those ≥ 2 years of age and < 10 years of age. ^†^Scoring system developed by the Brazilian National Ministry of Health (NMH) for diagnosing tuberculosis in children.

**Figure 2:**
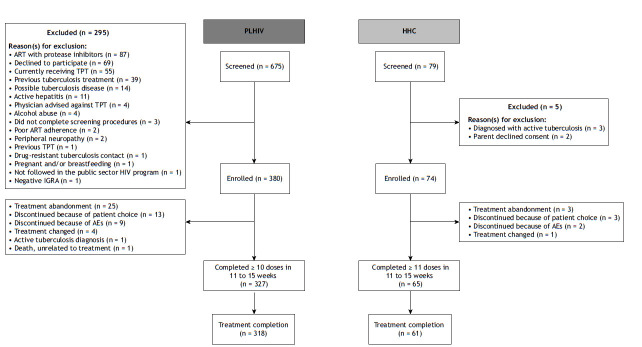
Study flow diagram. PLHIV: people living with HIV; HHCs: household contacts; TPT: tuberculosis preventive therapy; ART: antiretroviral therapy; IGRA: interferon-gamma release assay; and AEs: adverse events.

Eligible participants were identified, treated, and monitored in accordance with Brazilian national tuberculosis guidelines (Figures 1 and 2) at four public-sector health clinics. In the city of Rio de Janeiro, enrollment took place at three primary health care facilities: *Centro Municipal de Saúde Píndaro de Carvalho Rodrigues*, *Centro Municipal de Saúde João Barros Barreto*, and *Clínica da Família Rinaldo de Lamare*. In the city of Manaus, enrollment was overseen by the *Fundação de Medicina Tropical Doutor Heitor Vieira Dourado*, a tertiary health care facility specializing in infectious diseases. Inclusion and exclusion criteria were applied uniformly across sites. However, because of the clinical profile of the patients, the tertiary health care facility in Manaus may have enrolled participants with more advanced HIV or complex comorbidities. 

Adult PLHIV (≥ 18 years of age) were eligible if they were enrolled in public-sector HIV care; had no active tuberculosis; had not received tuberculosis treatment in the last two years; had never received TPT (unless newly exposed); and provided written informed consent. Exclusion criteria included inability to take oral medication; body weight < 30 kg; drug-resistant tuberculosis exposure in the last 12 months; isoniazid intolerance; grade 3/4 peripheral neuropathy; pregnancy/breastfeeding; substance abuse; and antiretroviral therapy with protease inhibitors, nevirapine, tenofovir alafenamide, or cobicistat. Pediatric HHCs (in the 2- to 14-year age bracket) were eligible if they were pulmonary tuberculosis contacts; had no history of current or past active tuberculosis disease; and had never received TPT (unless newly exposed and HIV positive). Exclusion criteria included inability to take oral medication; body weight < 10 kg; drug-resistant tuberculosis exposure; isoniazid intolerance; and antiretroviral therapy with dolutegravir, raltegravir, protease inhibitors, or nevirapine. 

According to Brazilian national tuberculosis guidelines, PLHIV with a CD4 count of ≤ 350 cells/mm^3^ or an unknown CD4 count are eligible for TPT after exclusion of active tuberculosis. For individuals with a CD4 count > 350 cells/mm^3^, TPT is recommended following exclusion of active tuberculosis and a positive result on either a tuberculin skin test or interferon-gamma release assay (Figure 1). 

In the present study, participants in the 10- to 18-year age bracket were considered to be adolescents. Asymptomatic HHCs ≥ 10 years of age are eligible for TPT after active tuberculosis is excluded; a positive tuberculin skin test result; and a normal chest X-ray. Symptomatic individuals undergo further evaluation: those with evidence of active tuberculosis are treated accordingly, whereas those with persistent symptoms without evidence of active tuberculosis require further investigation (Figure 1). 

For HHCs < 10 years of age, asymptomatic individuals are eligible for TPT after exclusion of active tuberculosis; a positive tuberculin skin test or interferon-gamma release assay; and a normal chest X-ray. Symptomatic children with evidence of active tuberculosis are treated for active disease, whereas those without active tuberculosis are further investigated using the Brazilian National Ministry of Health scoring system to guide management (Figure 1). 

PLHIV ≥ 18 years of age and HHCs in the 2- to 14-year age bracket who met eligibility criteria in accordance with Brazilian national guidelines were eligible for enrollment. Enrolled participants were prescribed 3HP at monthly clinic visits and monitored for tuberculosis signs and symptoms, adherence, and adverse events over the three-month treatment course. Vitamin B6 was concomitantly prescribed to reduce the risk of peripheral neuropathy. For adult PLHIV, the 3HP regimen consisted of 900 mg of isoniazid (three 300 mg tablets) and 900 mg of rifapentine (six 150 mg tablets), taken once weekly for 12 weeks with vitamin B6, totaling 10 tablets per dose. For pediatric HHCs, weight-based dosing was used. Rifapentine was prescribed at 300-750 mg (2-5 150 mg tablets) and isoniazid at 300-700 mg (via combinations of 100 mg and/or 300 mg tablets), administered once weekly for 12 weeks. To ascertain adherence, participants were provided with blister packs containing the exact number of doses required for the month. At each monthly visit, they were instructed to return the blister packs, allowing providers to verify the number of doses taken. Treatment completion was defined in accordance with national guidelines, as 12 weekly doses of 3HP taken in 12-15 weeks. Treatment interruption was defined as three missed doses (i.e., having taken ≤ 9 doses). Treatment discontinuation was categorized as being related to adverse events or patient choice. 

Follow-up of adult PLHIV was conducted at six months after treatment completion to assess tuberculosis outcomes through medical record reviews and data from the Brazilian National Ministry of Health Case Registry Database. 

De-identified patient-level data on treatment initiation, adverse events, and treatment completion were entered into a secure Research Electronic Data Capture (REDCap; Vanderbilt University, Nashville, TN, USA) database. Data were analyzed with Stata software, version 18.0 (StataCorp LLC, College Station, TX, USA). Data are presented as frequencies (percentages) for categorical variables and medians (interquartile ranges) for continuous variables. 

All study participants provided written informed consent. HHCs provided assent in addition to the written informed consent provided by their legal guardians. The study was conducted under a common protocol approved by the WHO Ethics Review Committee and was approved by the Johns Hopkins Medicine Institutional Review Board (Protocol no. IRB00258322), the Brazilian National Research Ethics Committee, and relevant local institutional review boards. 

## RESULTS

From October of 2021 to March of 2023, 380 PLHIV and 74 HHCs were enrolled in the study and initiated TPT with 3HP (Figure 2). 

### Demographics of study participants

#### PLHIV

A total of 380 PLHIV were enrolled: 243 in the city of Rio de Janeiro and 137 in the city of Manaus (Table 1). Most of the study participants were male, accounting for 77.6% of the sample, with similar proportions observed in Rio de Janeiro (78.2%) and Manaus (76.6%). The median age of PLHIV was 40 years (IQR, 31-51), with slightly higher median ages in Rio de Janeiro (41 years; IQR, 32-52) than in Manaus (36 years; IQR, 28-47). The median BMI was 25.4 kg/m^2^ (IQR, 22.7-28.6), with comparable values in Rio de Janeiro (25.7 kg/m^2^; IQR, 23.2-28.8) and Manaus (24.6 kg/m^2^; IQR, 21.9-27.6). Most (80.8%) of the PLHIV were on dolutegravir-based antiretroviral therapy at enrollment (83.5% in Rio de Janeiro and 75.9% in Manaus). 

**Table 1:** Baseline characteristics of patients receiving tuberculosis preventive therapy with three months of once-weekly isoniazid plus rifapentine.^a^

	Overall	Rio de Janeiro	Manaus
	PLHIV (N = 380)	PLHIV (n = 243)	PLHIV (n = 137)
Male	295 (77.6)	190 (78.2)	105 (76.6)
Age, years	40 [31-51]	41 [32-52]	36 [28-47]
BMI, kg/m^2^	25.4 [22.7-28.6]	25.7 [23.2-28.8]	24.6 [21.9-27.6]
Dolutegravir-based ART	307 (80.8)	203 (83.5)	104 (75.9)
	HHCs (N = 74)	HHCs (n = 27)	HHCs (n = 47)
Male	40 (54.1)	15 (55.6)	25 (53.2)
Age, years	8.6 [5.1-11.8]	8.6 [4.9-12.6]	9.1 [5.1-11.8]
Normal weight	65 (87.8)	27 (100)	38 (80.9)
Weight-for-age Z-score < −2	9 (12.2)	0	9 (19.1)

PLHIV: people living with HIV; ART: antiretroviral therapy; and HHCs: household contacts. ^a^Data presented as n (%) or median [IQR].

#### HHCs in the 2- to 14-year age bracket

Seventy-four HHCs were enrolled: 27 in Rio de Janeiro and 47 in Manaus. Of those, 54.1% were male, with similar proportions in Rio de Janeiro (55.6%) and Manaus (53.2%). The median age of HHCs was 8.6 years (IQR, 5.1-11.8), being 8.6 years in Rio de Janeiro (IQR, 4.9-12.6) and 9.1 years in Manaus (IQR, 5.1-11.8). All of the participants in Rio de Janeiro had normal weight; undernutrition or stunting was present in 19.1% of the participants in Manaus. 

### Treatment outcomes

#### PLHIV

The treatment completion rate in accordance with Brazilian national tuberculosis guidelines was 83.7%, with Rio de Janeiro achieving a slightly higher completion rate of 86.0%, in comparison with 79.6% in Manaus (Table 2). Of the study participants, 86.0% completed 10 doses within an 11 to 15-week time frame, with comparable completion rates in Rio de Janeiro (86.4%) and Manaus (86.1%). 

**Table 2:** Treatment outcomes in people living with HIV and household contacts in the 2- to 14-year age bracket receiving tuberculosis preventive therapy with three months of once-weekly isoniazid plus rifapentine.^a^

	Overall	Rio de Janeiro	Manaus
	PLHIV (N = 380)	PLHIV (n = 243)	PLHIV (n = 137)
Treatment completion in accordance with Brazilian national tuberculosis guidelines	318 (83.7)	209 (86.0)	109 (79.6)
Completed ≥ 11 doses in 11 to 15 weeks	324 (85.3)	210 (86.4)	114 (83.2)
Completed ≥ 10 doses in 11 to 15 weeks	327 (86.0)	210 (86.4)	118 (86.1)
Discontinued because of patient choice	13 (3.4)	5 (2.1)	8 (5.8)
Discontinued because of adverse events	9 (2.4)	8 (3.3)	1 (0.7)
Treatment changed	4 (1.1)	2 (0.8)	2 (1.5)
Treatment interruption	25 (6.6)	18 (7.4)	7 (5.1)
Active tuberculosis diagnosis	1 (0.3)	0	1 (0.7)
Death^b^	1 (0.3)	0	1 (0.7)
	HHCs (N = 74)	HHCs (n = 27)	HHCs (n = 47)
Treatment completion in accordance with Brazilian national tuberculosis guidelines	61 (82.4)	23 (85.2)	38 (80.9)
Completed ≥ 11 doses in 11 to 15 weeks	65 (87.8)	25 (92.6)	40 (85.1)
Discontinued because of patient choice	3 (4.1)	1 (3.7)	2 (4.3)
Discontinued because of adverse events	2 (2.7)	1 (3.7)	1 (2.1)
Treatment changed	1 (1.4)	0	1 (2.1)
Treatment interruption	3 (4.1)	0	3 (6.4)
Active tuberculosis diagnosis	0	0	0

PLHIV: people living with HIV; and HHCs: household contacts. ^a^Data presented as n (%). ^b^Death unrelated to treatment, caused by disseminated cryptococcosis.

Treatment discontinuation due to patient choice occurred in 3.4% of participants, with a higher discontinuation rate in Manaus (5.8%) than in Rio de Janeiro (2.1%). Adverse events led to treatment discontinuation in 2.4% of participants, with most cases occurring in Rio de Janeiro (3.3%) and one case in Manaus (0.7%). 

Treatment was changed for 1.1% of participants, with two participants each in Rio de Janeiro (0.8%) and Manaus (1.5%). Treatment interruption occurred in 6.6% of participants, being slightly higher in Rio de Janeiro (7.4%) than in Manaus (5.1%). 

Active tuberculosis was diagnosed in one participant (0.3%) during 3HP treatment in Manaus, where one death unrelated to 3HP was also reported (0.7%). No active tuberculosis diagnoses or deaths were recorded among participants in Rio de Janeiro. 

#### HHCs in the 2- to 14-year age bracket

Of the HHCs in the present study, 82.4% completed treatment, with a slightly higher completion rate in Rio de Janeiro (85.2%) than in Manaus (80.9%). In addition, 87.8% of participants completed 11 doses within an 11 to 15-week period, with a higher rate in Rio de Janeiro (92.6%) than in Manaus (85.1%). 

Treatment was discontinued by patient choice in 4.1%, with one participant in Rio de Janeiro (3.7%) and two in Manaus (4.3%). Adverse events led to treatment discontinuation in 2.7% of HHCs, with one case each in Rio de Janeiro (3.7%) and Manaus (2.1%). 

Treatment was changed for 1.4% of HHCs, all of whom were in Manaus (2.1%), whereas no changes were recorded in Rio de Janeiro. Additionally, treatment interruption occurred in 4.1% of cases, exclusively in Manaus (6.4%), with no occurrences in Rio de Janeiro. 

No active tuberculosis diagnoses occurred among the HHCs while on treatment. 

### Tolerability

Treatment discontinuation because of toxicity was uncommon in both groups of participants (Table 3). Among PLHIV, systemic hypersensitivity affected 0.8%, all of whom were in Rio de Janeiro (1.2%). Mild hepatotoxicity and flu-like reactions accounted for discontinuation in 0.5% of participants, again limited to those in Rio de Janeiro (0.8%). Additionally, one participant in Rio de Janeiro discontinued treatment because of peripheral neuropathy (0.3% overall; 0.4% in Rio de Janeiro). In Manaus, one participant discontinued treatment because of nausea and vomiting (0.7%). 

**Table 3:** Treatment-discontinuing adverse events in people living with HIV and household contacts in the 2- to 14-year age bracket receiving tuberculosis preventive therapy with three months of once-weekly isoniazid plus rifapentine.^a^

Treatment-discontinuing AEs	Overall	Rio de Janeiro	Manaus
	PLHIV (N = 380)	PLHIV (n = 243)	PLHIV (n = 137)
Flu-like reaction	2 (0.5)	2 (0.8)	0
Mild hepatotoxicity	2 (0.5)	2 (0.8)	0
Systemic hypersensitivity	3 (0.8)	3 (1.2)	0
Peripheral neuropathy	1 (0.3)	1 (0.4)	0
Other^b^	1 (0.3)	0	1 (0.7)
	HHCs (N = 74)	HHCs (n = 27)	HHCs (n = 47)
Peripheral neuropathy	1 (0.1)	0	1 (2.1)
Other^c^	1 (0.1)	1 (0.4)	0

AEs: adverse events; PLHIV: people living with HIV; and HHCs: household contacts. ^a^Data presented as n (%). ^b^Nausea and vomiting. ^c^Vomiting.

Peripheral neuropathy led to discontinuation in one HHC in Manaus (2.1%), whereas vomiting was the cause of discontinuation for one HHC in Rio de Janeiro (0.4%). 

### Follow-up

Six-month follow-up after treatment completion was performed for 271 of the 380 PLHIV enrolled in the present study. At six months, 99.6% of the 271 PLHIV remained free of tuberculosis, with all of the participants in Rio de Janeiro (100%) and most of those in Manaus (98.7%) showing no signs of active tuberculosis. There was one reported death unrelated to tuberculosis or 3HP in Manaus (1.3%), whereas no deaths were recorded among the participants in Rio de Janeiro (Table 4). 

**Table 4:** Six-month follow-up outcomes in people living with HIV and receiving tuberculosis preventive therapy with three months of once-weekly isoniazid plus rifapentine.^a^

Outcome at follow-up	Overall	Rio de Janeiro	Manaus
	N = 271	n = 194	n = 77
Free of tuberculosis	270 (99.6)	194 (100)	76 (98.7)
Death^b^	1 (3.7)	0	1 (1.3)

a Data presented as n (%). ^b^Death caused by dissecting aortic aneurysm.

## DISCUSSION

The implementation of the 3HP regimen for TPT in Brazil, specifically among PLHIV and HHCs, has shown promising results in terms of treatment completion rates and overall tolerability. The high completion rates in our study-83.7% among PLHIV and 82.4% among HHCs-demonstrate that the 3HP regimen is both feasible and acceptable in real-world settings, supporting its broader implementation in tuberculosis control programs. Adverse events leading to treatment discontinuation were minimal, emphasizing the overall tolerability of 3HP (PLHIV: 2.4%; HHCs: 2.7%). These outcomes are consistent with the literature regarding the robust safety profile of 3HP, underscoring that 3HP is a feasible and well-tolerated option for tuberculosis prevention in these vulnerable populations. However, differences in outcomes between Rio de Janeiro and Manaus highlight contextual factors that may influence TPT completion and tolerability across different settings. The higher completion rates observed in Rio de Janeiro, particularly among PLHIV (PLHIV: 86.0% vs. 79.6%; HHCs: 85.2% vs. 80.9%), may reflect differences in health care infrastructure and participant complexity between the two types of health care settings. In Rio de Janeiro, care was provided at primary health care facilities, offering more geographically accessible, community-based routine and preventive care. In Manaus, however, care was provided at a tertiary health care facility, serving a larger, geographically dispersed population possibly facing greater barriers to accessing care-including longer travel times for medical care and follow-up-and more complex conditions, such as lower CD4 counts.^19^ Poor transportation infrastructure, particularly in northern Brazil, where Manaus is located, amplifies health care access challenges. Studies show that geographic and systemic inequities within the Brazilian public health care system drive regional disparities in health care access and quality across various services.^20^ ^,^ ^21^ Although contextual factors likely influenced site differences in the present study, small sample sizes could exaggerate percentage differences in treatment discontinuation and interruption, requiring cautious interpretation. 

Although the completion rates for ≥ 10 doses among PLHIV in Rio de Janeiro and Manaus were similar (86.4% vs. 86.1%), they do not represent the complete 12-dose regimen. This means that although many patients were close to completing the entire course, a gap remains to be addressed. If patients drop out after 10 or 11 doses, they may not receive the full protective benefit of the regimen, potentially leaving them at risk for developing active tuberculosis. Although adherence to 10 or more doses is not categorized as treatment interruption by Brazilian national or WHO guidelines, the data suggest that additional efforts should be made to ensure that patients complete the 12-dose regimen. This might involve improved follow-up, patient education, and support services to address barriers that might prevent patients from completing the regimen and reduce the risk of developing active tuberculosis. The high pill burden of the 10-tablet weekly dose may have contributed to decreased adherence, underscoring the importance of considering fixed-dose combination formulations during scale-up. Notably, Brazil began distributing the fixed-dose combination formulation of 3HP in February of 2024,^22^ possibly enhancing tolerability and adherence among those receiving 3HP and ultimately supporting better treatment completion and maximizing the protective benefits of the regimen. 

Pediatric populations face unique challenges. The use of nondispersible tablets presents challenges such as difficulty swallowing, poor palatability, and caregiver burden, all of which may affect adherence and limit scale-up in children.^23^ ^,^ ^24^ Although 74 pediatric HHCs were included in the present study, further studies are warranted to assess 3HP performance in larger cohorts of children and adolescents and to evaluate child-friendly regimens, such as dispersible tablets, to ensure equitable access to TPT across age groups. 

The findings from the six-month follow-up of PLHIV in the present study underscore the high efficacy of the intervention in preventing tuberculosis among PLHIV, with all but one participant who died of causes unrelated to tuberculosis maintaining a tuberculosis-free status at the six-month mark. 

Brazilian national reports indicate that 71.6% of patients completed 3HP from rollout to April of 2022, and 1.3% discontinued 3HP because of adverse events.^3^ The national data are consistent with the trends observed in our study, reinforcing the importance of ongoing support and monitoring to maintain high adherence levels and minimize treatment interruption. The agreement between our findings and national reports strengthens the case for a broader implementation of the 3HP regimen, with an emphasis on overcoming regional disparities. 

The strengths of the present study include its multicenter design and the use of a standardized regimen across diverse settings, allowing a comparison of outcomes between different regions. However, our study is not without limitations. The observational nature of the study and the lack of a control group limit the ability to draw causal inferences about the factors contributing to the differences in outcomes between sites. Local factors, such as health care infrastructure, environmental conditions, and patient demographics, may play a significant role in influencing outcomes, and these factors should be further explored in future studies. The six-month follow-up period may not have captured long-term protection; extended monitoring could improve evaluation of outcomes and recurrence risk. 

Despite the positive intent of the policy change, regulatory delays in the importation of rifapentine emerged as a critical barrier to the timely implementation of 3HP. The challenges during this period shed light on the bureaucracy involved in translating policy change into effective practice. Understanding and addressing such barriers are essential for future policy implementations. 

In April of 2023, the Brazilian government introduced the Interministerial Committee for the Elimination of Tuberculosis and Other Socially Determined Diseases, which is aimed at fostering multisectoral collaboration to eliminate tuberculosis in Brazil.^25^ ^,^ ^26^ This initiative, including the Brazilian National Ministry of Health and the eight other federal ministries, set ambitious targets to reduce the incidence of tuberculosis to fewer than 10 cases per 100,000 population and the annual number of tuberculosis deaths to fewer than 230 by 2023. Our findings support the ongoing implementation of the 3HP regimen as a vital component in achieving these goals, emphasizing the need for continued efforts to overcome regulatory and logistical barriers that could hinder progress. 

The findings of the present study provide valuable insights into the practical aspects of translating policy changes into effective tuberculosis control strategies. Despite initial regulatory delays, 3HP was successfully introduced, showed high treatment completion rates, and was well tolerated, constituting a promising strategy for accelerating tuberculosis elimination in Brazil. The present study demonstrates that short-course TPT can be effectively integrated into national tuberculosis control programs, achieving high rates of treatment completion and low rates of treatment-discontinuing adverse events. As Brazil continues its efforts to combat tuberculosis, the success of 3HP implementation provides a compelling case for a broader adoption of short-course TPT regimens to accelerate progress toward tuberculosis elimination. 

## References

[1] World Health Organization (WHO) [homepage on the Internet] (c2024). Global tuberculosis report 2024.

[2] World Health Organization (2021). WHO global lists of high-burden countries for tuberculosis (TB), TB/HIV, and multidrug/rifampicin-resistant TB (MDR/RR-TB), 2021-2025: background document. Geneva,.

[3] Ministério da Saúde (c2024). Boletim Epidemiológico de Tuberculose - Número Especial.

[4] Gunasekera KS, Vonasek B, Oliwa J, Triasih R, Lancioni C, Graham SM (2022). Diagnostic challenges in childhood pulmonary tuberculosis-optimizing the clinical approach. Pathogens.

[5] Maphalle LNF, Michniak-Kohn BB, Ogunrombi MO, Adeleke OA (2022). Pediatric tuberculosis management a global challenge or breakthrough?. Children (Basel).

[6] Whittaker E, López-Varela E, Broderick C, Seddon JA (2019). Examining the complex relationship between tuberculosis and other infectious diseases in children. Front Pediatr.

[7] Hamada Y, Getahun H, Tadesse BT, Ford N (2021). HIV-associated tuberculosis. Int J STD AIDS.

[8] Pinto PFPS, Teixeira CSS, Ichihara MY, Rasella D, Nery JS, Sena SOL (2024). Incidence and risk factors of tuberculosis among 420,854 household contacts of patients with tuberculosis in the 100 Million Brazilian Cohort (2004-18) a cohort study. Lancet Infect Dis.

[9] Dye C, Glaziou P, Floyd K, Raviglione M (2013). Prospects for tuberculosis elimination. Annu Rev Public Health.

[10] World Health Organization (2023). Global tuberculosis report, 2023..

[11] Alsdurf H, Hill PC, Matteelli A, Getahun H, Menzies D (2016). The cascade of care in diagnosis and treatment of latent tuberculosis infection a systematic review and meta-analysis. Lancet Infect Dis.

[12] Kendall EA, Durovni B, Martinson NA, Cavalcante S, Masonoke K, Saraceni V (2020). Adherence to tuberculosis preventive therapy measured by urine metabolite testing among people with HIV. AIDS.

[13] Stuurman AL, Vonk Noordegraaf-Schouten M, van Kessel F, Oordt-Speets AM, Sandgren A, van der Werf MJ (2016). Interventions for improving adherence to treatment for latent tuberculosis infection A systematic review. BMC Infect Dis.

[14] World Health Organization (2018). Latent tuberculosis infection: updated and consolidated guidelines for programmatic management. Geneva,.

[15] Sterling TR, Villarino ME, Borisov AS, Shang N, Gordin F, Bliven-Sizemore E (2011). Three months of rifapentine and isoniazid for latent tuberculosis infection. N Engl J Med.

[16] Salazar-Austin N, Dowdy DW, Chaisson RE, Golub JE (2019). Seventy Years of Tuberculosis Prevention Efficacy, Effectiveness, Toxicity, Durability, and Duration. Am J Epidemiol.

[17] World Health Organization (2022). WHO Consolidated Guidelines on Tuberculosis..

[18] Brasil (2021). Ministério da Saúde. Nota Informativa No 5/2021-CGDR/DCCI/SVS/MS - Departamento de HIV, Aids, Tuberculose, Hepatites Virais e Infecções Sexualmente Transmissíveis.

[19] Vicari T, Lago LM, Bulgarelli AF (2022). Realities of the practices of the Family Health Strategy as driving forces for access to SUS health services a perspective of the Institutional Analysis. Saude Debate.

[20] Gómez EJ, Jungmann S, Lima AS (2018). Resource allocations and disparities in the Brazilian health care system insights from organ transplantation services. BMC Health Serv Res.

[21] Miranda ALDC, Costa VLDS, da Paixão ART, Martins MB, Polaro SHI, Cunha CLF (2024). Factors associated with access to health services among people with long COVID in the Brazilian Amazon. Front Public Health.

[22] Brasil (2024). Ministério da Saúde. Nota Informativa No 2/2024-CGTM/DATHI/SVSA/MS - Departamento de HIV, Aids, Tuberculose, Hepatites Virais e Infecções Sexualmente Transmissíveis.

[23] Golhen K, Buettcher M, Kost J, Huwyler J, Pfister M (2023). Meeting challenges of pediatric drug delivery The potential of orally fast disintegrating tablets for infants and children. Pharmaceutics.

[24] Khatri A, Verma M, Patidar AB, Kumari P, Akram H (2025). Evaluating the challenges of oral drug administration in children a review. Int J Contemp Pediatr.

[25] Maciel ELN, Sanchez MN, Cruz AMD, Cravo DB, Lima NVT (2024). Brazil's pivotal moment in public health establishing the Interministerial Committee (CIEDDS) for the elimination of tuberculosis and socially determined diseases. Rev Soc Bras Med Trop.

[26] Brasil (2023). Imprensa Nacional. Decreto No 11.494, de 17 de abril de 2023. DOU Ed. 74, Seção 1, p.14.

